# The Histology of the Conscience

**Published:** 1891-09-12

**Authors:** 


### The Hospital

A Weekly Journal of

Science, Medicine, Nursing, and Philanthropy.

*For Hospitals, Asylums, Infirmaries, Dispensaries, Medical Practitioners, Students, Nurses, and the Charitable Public.*

Vol. X.—No. 259.] [Sept. 12, 1891.

### The Histology of the Conscience.

The medical students of the present day are for the most part men, and men of education. Unlike their predecessors, they no longer confine their extra-professional studies to such subjects as beer and rat-killing. Mr. Robert Sawyer—peace to his bones—did not know anything about histology of any kind: the idea of a histological conscience would have appeared to him as natural as that of a mathematical elephant. Is the conscience, like the liver and the cardiac valves, an integral and essential part of the entity called "man"? This, it will be perceived, is a fundamental question; and the consideration of it is quite as interesting to the anthropologist and the scientific doctor as it is to the theologian and the metaphysician.

The conscience is as much a part of the normal human being, as the nervous system, and it has probably been developed on similar lines. In the lowest forms of animal life there is no demonstrable nervous system. But it is probable that in all organisms which possess sensibility to impressions, and the power of contractility, there is a rudimentary system of nerves if we could but get at it. We know, at any rate, that in animals which are but a step removed from the lowest there are nerves which receive external impressions and produce reflex motions; in other words there are sensory and motor nerves. From these as a beginning, there is developed every conceivable variety of nervous system up to the brain and chord and trunk and peripheral nerves of a Mendelsshon, a Faraday, a Kant, and a Shakespeare. Without a nervous system man would not be man, as we know him.

Can there be such a thing as a rudimentary conscience on the one hand, and a developed conscience on the other, which may be compared respectively with a rudimentary and a highly developed nervous system? Undoubtedly there can. We will go a step further and say—there is, and must be. What is the function of the conscience? In its most rudimentary condition it simply says—"I ought," or "I ought not." That is its function, to recognise duty; the duty of doing, or the duty of refraining. It is a circumstance to be soberly weighed by scientific men that what is called by theologians "the fall of man" was really the suddenly perceived fact of moral distinctions. According to the tradition recorded in the book of Genesis, the tempter offered to the woman the knowledge of good and evil. Presumably, therefore, there had not been up to that time any knowledge of moral distinctions in the human world. That is to say, so far as the fact of morality was concerned, man was then in the same condition as the ape, or the horse, or the dog of the present day. Whatever be the scientific value of the story of Adam and Eve's temptation, it is certain that at some period in the history of the race, man became conscious of duty. "Thou shalt," and "thou shalt not," rang through the chambers of his being in tones of authority which he felt he could not dispute.

That was the rudimentary conscience, a kind of histologically simple differentiation from the spiritual protoplasm of man's nature. It was a faculty which received and responded to impressions, exactly as the rudimentary nervous system of the lowest animals receives external stimuli, and responds to them by contractility. It is obvious that there are many kinds of stimuli to which the rudimentary nervous system makes no manner of response. The musician with his most ravishing music, appeals in vain to the protozoon. A Demosthenes, a Chrysostom, or a Burke, would be powerless to carry even the faintest conviction to the nerve organs of an Echinoderm. Aristotle's lantern shines with no philosophic light, and Venus' flower basket is destitute of the consciousness of its own beauty. Yet it is through such stages as these that the nervous system has passed on its painful and arduous journey to the regions which yield their knowledge to the astronomer's brain. Every form of stimulus in the universe finds a response in some man or woman's cerebrum.

It is exactly so with the conscience. According to the story of Genesis, the first prohibition laid upon the developing moral faculty was: "Thou shalt not eat"—an apple. An incredible story, according to the shallow philosophy of the critic who writes before he has learnt to think. But a story which admirably illustrates the contention that affirms a rudimentary conscience; a conscience which at first responded to as strictly limited a number of stimuli as does the nervous system of the protozoon.

At what stage of development has the conscience of the medical student arrived? It cannot be rudimentary, except on the assumption of an intellectual retrograde metamorphosis. The civilised man's conscience has passed through all the stages of development of the nervous system, and it is now, when in its normal condition, as capable of responding to an almost infinite number of stimuli. The highest and the greatest variety of nobleness in the world must appeal to the highest minds among medical students with the authority of commands; and the darkest evil must similarly produce a response of repulsion and hatred.

In dealing with the conscience we are dealing with an integral and essential part of man's nature. Without a conscience, as without a nervous system, man is not man; and without a highly specialised conscience any individual man must be held to be at a low stage of development. The brain and the nervous system consist of a comparatively simple tissue highly specialised in civilised and cultured men. Similarly the simple conscience, with its elementary "thou shalt" and "thou shalt not," has been specialised until the greatest and noblest of men live their God-like lives almost with the ease and naturalness of instinct.

Medical students are preparing themselves for a kind of scientific priesthood. It is given to them to see more deeply than others into some of the ultimate facts of human nature. Knowledge, and the faculty for gaining more knowledge, bring corresponding obligations. An intelligent student of medicine cannot be reckless and vicious. The various light that streams in upon his intellect touches the spreading fibrils of his conscience at a thousand points, and rouses it to affirmation or denial. On the positive side he sees new and almost unsuspected regions of knowledge opening out before him, and his conscience, with whip and spur, urges him to take full possession. Loafing, drinking, gambling, the indulgence of the lower passions—these are impossible to the student who is worthy of the name. Some of the older forms and demands of religion may have lost their authority over him. But a new faith gradually takes possession of his soul; the passion for knowledge, for truth, for power to bind and to loose in those mysterious regions in which pain and sorrow play their tragic parts. Hatred is often one of the noblest and most righteous of passions. The worthy medical student hates loafing and loafers. Such fools are they; such brute beasts, and worse than brute beasts; so coarse are their passions, so low and vile their thoughts and ways; so worse than worthless are they in character, in conduct, and in reputation that he loaths the sight of them. The medical schools are purging themselves more and more of the idle, the vicious, and the stupid. The time is coming when medicine, which is now so patiently searching out all that can be known of man and of his whole race, shall be the great teacher to point out to him both his duty and his destiny as the highest of all God's creatures.

